# Importance of Susceptibility Rate of ‘the First’ Isolate: Evidence of Real-World Data

**DOI:** 10.3390/medicina56100507

**Published:** 2020-09-28

**Authors:** Sollip Kim, Soo Jin Yoo, Jeonghyun Chang

**Affiliations:** 1 Laboratory Medicine, Inje University, Ilsan Paik Hospital, Goyang 10380, Korea; lalacopine@gmail.com; 2 Laboratory Medicine, Inje University, Sanggye Paik Hospital, Seoul 10757, Korea; sjyoo@paik.ac.kr

**Keywords:** cumulative antibiogram, susceptibility, first isolate, empirical therapy, antimicrobial agent

## Abstract

*Background and objectives:* For proper antimicrobial therapy, cumulative antibiograms should be representative of geographic region and be accurate. Clinical and Laboratory Standards Institute (CLSI) guidelines recommend that only the first isolates (FI) of a species per patient are used when reporting cumulative antibiograms. However, >50% of hospitals in the United States report antibiograms of all isolates. We compared antibiograms from the FI with those from total isolates (TI). *Materials and Methods:* Antimicrobial data of all isolates identified in the Microbiology unit of Ilsan Paik Hospital in 2019 were retrospectively acquired from the hospital information system. The susceptibility rates to antimicrobials of *Escherichia coli*, *Acinetobacter baumannii*, *Klebsiella pneumoniae*, *Pseudomonas aeruginosa*, *Staphylococcus aureus*, *Enterococcus faecium*, and *Enterococcus faecalis* were analyzed by FI and TI, respectively. Isolate counts and susceptibility rates of each species for the reported antimicrobials were compared. *Results:* The numbers of isolates by FI/TI were as follows: 1824/2692 *E. coli*, 480/1611 *A. baumannii*, and 662/1306 *K. pneumoniae*, and 407/953 *P. aeruginosa* for gram-negative bacteria and 649/1364 *S. aureus*, 211/313 *E. faecium*, and 323/394 *E. faecalis* for gram-positive bacteria. All antimicrobial agents showed higher susceptibility rates when calculated as FI than as TI in gram-negative bacteria except colistin: 3.7% for *E. coli*, 14.5% for *A. baumannii*, 8.3% for *K. pneumoniae*, and 7.9% for *P. aeruginosa*. In *S. aureus*, 8/11 antimicrobial agents revealed higher susceptibility rates for FI than for TI. *E. faecalis* and *E. faecium* showed lower susceptibility rates for 7/10 antimicrobial agents for FI than for TI. The oxacillin susceptibility rates of *S. aureus* were 36.6%/30.2% with FI/TI and vancomycin susceptibility rates for *E. faecium* were 54.1% and 49.5%, respectively. *Conclusions:* When comparing cumulative antibiograms by FI with TI using real-world data, there is a large gap for critical species requiring hospital infection control. Although FI calculation is difficult, antibiograms must be calculated as FI for proper preemptive antimicrobial therapy because FI provides proper antimicrobial susceptibility data.

## 1. Introduction

Inappropriate empirical antimicrobial therapy can lead to increased resistance to antimicrobial agents or ineffective treatment. The rates of antimicrobial susceptibilities of pathogens vary geographically. A cumulative antibiogram report in the hospital is most often used to guide initial empirical antimicrobial therapy to manage infections in patients who have not received definitive microbiological results to enable target treatment. Clinicians must understand the resistance rates of clinical isolates in local populations to ensure efficient and successful empirical treatment. Clinicians may treat patients with inappropriate empirical antimicrobial therapy using broad-spectrum antibiotic agents based on their inappropriate cumulative antibiograms, calculated based on total isolates, which can result in increased resistance to antimicrobial agents or ineffective treatment [1,2,3]. The Clinical and Laboratory Standards Institute (CLSI) guideline M39-A4 recommends that only the first isolate of a given species per patient, per analysis period, should be included in cumulative antibiogram reporting, irrespective of the body site, antimicrobial susceptibility profile, or other phenotypic characteristics when reporting cumulative antibiograms [4]. However, many laboratories still report antimicrobial susceptibility data based on total isolates rather than on the first isolate of patients. In a study of the preparation of cumulative antibiogram reports in the United States, only 38% of community hospitals stated that duplicate isolates were excluded from the report [5].

The reason for the calculation of the cumulative antibiogram using the total number of isolates is that clinicians working in microbiology laboratories may not be aware of the differences in the two results. Therefore, in this study, we evaluated the importance of calculating the cumulative antibiogram using the first isolate by analyzing actual clinical data to reveal the susceptibility differences between the two methods.

## 2. Materials and Methods

### 2.1. Study Environment

Susceptibility data used in this study were acquired from microbiological reports of Ilsan Paik Hospital. The Ilsan Paik Hospital is a 670-bed secondary care university hospital in a Seoul Metropolitan Area of Korea. This hospital has a pediatric department and maternity departments, including obstetrics, gynecology, and a neonatal department. There is a cancer center which has a medical oncology department, a radiation oncology department, and a surgery department for oncology patients, and a cardiovascular center for patients with acute coronary disease. The average number of hospitalization days in this hospital was 8.2 in 2019. In the clinical microbiology laboratory, identification and antimicrobial susceptibility tests of clinical isolates are performed by matrix-assisted laser desorption/ionization time-of-flight mass spectrometry using a Vitek MS (bioMérieux, Marcy-L’Etoile, France) and Vitek 2 automated identification and antimicrobial susceptibility system (bioMérieux) with the broth microdilution method and disk diffusion method as described in the CLSI guidelines [6]. The number of bacterial cultures ordered for clinical specimens was 66,051 in 2019.

### 2.2. Data Collection

The most frequently isolated organisms, such as *Escherichia coli*, *Klebsiella pneumoniae*, *Acinetobacter baumannii*, *Pseudomonas aeruginosa*, *Staphylococcus aureus*, *Enterococcus faecalis*, and *Enterococcus faecium* were included in the analysis. Antimicrobial susceptibility data of clinical isolates identified in the clinical microbiology laboratories in 2019 were retrospectively collected from the hospital information system. Antimicrobial agents used in the study were listed in Table 1. The list of antimicrobial agents reported was based on the CLSI guidelines [4] and clinicians’ opinions. Isolate identification at the species level, patients’ medical record number, and antimicrobial susceptibility results of each isolate were acquired.

### 2.3. Definition of Two Groups: FI and TI

The FI (first isolate) was defined as the first isolate of a given species per patient, per analysis period from 1 January 2019 to 31 December 2019, which was included regardless of the specimen collection site in the body, antimicrobial susceptibility profile, or other phenotypic characteristics. The TI (total isolate) was defined as the total number of identified isolate per analysis period from 1 January 2019 to 31 December 2019. Data were analyzed for both groups. The WHONET 5.6 program was used for grouping and further analysis (available from: URL: https://www.who.int/medicines/areas/rational_use/AMR_WHONET_SOFTWARE/en/) [7].

### 2.4. Susceptibility Rate Analysis by FI and TI

The susceptibility rates of each reported antimicrobial agent were calculated by both FI and TI, and the results were compared. We analyzed differences in the susceptibility rates obtained by FI or TI. The mean of the gaps in susceptibility rates between FI and TI among the reported antimicrobial agents per study organism was calculated.

### 2.5. Important Multi-Drug Resistant Organism by FI and TI

The prevalence of carbapenem-resistant *E. coli*, carbapenem-resistant *K. pneumoniae*, carbapenem-resistant *A. baumannii*, carbapenem-resistant *P. aeruginosa*, methicillin-resistant *S. aureus*, and vancomycin-resistant *Enterococcus* of obtained by FI and TI was compared. For vancomycin-resistant *Enterococcus*, only *E. faecium* was included because of the low prevalence of vancomycin-resistance in *E. faecalis* [8]. These prevalences were calculated using WHONET 5.6 program was used for grouping and further analysis (available from: URL: https://www.who.int/medicines/areas/rational_use/AMR_WHONET_SOFTWARE/en/).

### 2.6. Statistical Analysis

The proportions of the susceptibility rates of each antimicrobial agent and prevalence of multidrug-resistant organisms by FI and TI were compared using a Chi-squared test for the comparison of two proportions in MedCalc Version 17.9.7 (MedCalc Software, Ostend, Belgium). A P-value of less than 0.05 was considered as statistically significant.

## 3. Results

### 3.1. Number of Isolates and TI/FI Ratio

During the study period, a total of 12,553 isolates were identified by TI, and 7533 isolates were identified by FI and reported in our clinical microbiology laboratory. The number of study organisms and proportions of TI/FI were as follows: 2692/1824 (1.5) for *E. coli*, 1611/480 (3.4) for *A. baumannii*, 1306/662 (2.0) for *K. pneumoniae*, 953/407 (2.3) for *P. aeruginosa*, 1364/649 (2.1) for *S. aureus*, 394/323 (1.2) for *E. faecalis*, and 313/211 (1.5) for *E. faecium* (Table 2).

### 3.2. Susceptibility Rates

In gram-negative organisms, all antimicrobial susceptibility rates of *E. coli*, *K. pneumoniae*, and *P. aeruginosa*, except colistin, of *A. baumannii* were higher according to FI than to TI. Differences in the susceptibility rate between FI and TI were significant in 13/15 antimicrobial agents for *E. coli*, 12/15 for *A. baumannii*, 12/14 for *K. pneumoniae*, and 8/11 for *P. aeruginosa* (Table 2). In *S. aureus*, 8 of 11 antimicrobial agents showed a higher susceptibility rate to FI than by TI, 6 of which were significant. Additionally, 3/10 *E. faecalis* and 3/10 *E. faecium* showed higher susceptibility rates according to FI than to TI, with no significant difference observed between FI and TI (Table 3) (Supplementary Materials).

### 3.3. Prevalence of Multi-Drug Resistant Organisms

The rates of multi-drug resistant organisms, according to FI and TI are listed in Table 3. The rates of multi-drug resistance of the organisms calculated by FI were lower than those calculated by TI. Carbapenem-resistant *K. pneumoniae*, carbapenem-resistant *A. baumannii*, carbapenem-resistant *P. aeruginosa*, and methicillin-resistant *S. aureus* showed significant differences in the values calculated using the two different methods (*p* < 0.05) (Table 4).

## 4. Discussion

Cumulative antibiograms play an important role in both hospitals and communities for monitoring local antibiograms and determining infection control strategies as well as empiric antibiotic usage policies [9,10]. The Infectious Diseases Society of America/Society for Healthcare Epidemiology of America antibiotic stewardship program guidelines recommend using cumulative antibiograms to guide the establishment of empiric therapy treatment guidelines [11]. Using accurate antibiograms in the local environment, an antibacterial de-escalation approach can be easily made to provide appropriate antimicrobial agents while minimizing the emergence of resistance [12]. In the community, antibiogram data can be used to monitor public health threats, such as infectious disease outbreaks involving antimicrobial-resistant pathogens [9].

For successful empirical antimicrobial treatment, it is important to determine the resistance profile of local communities and facilities. A cumulative antibiogram that appropriately reflects the local environment is necessary to achieve this goal. We analyzed and probed the differences in susceptibility rates between calculation by TI and FI using actual clinical data. Particularly, we confirmed that the susceptibility rates determined using TI are lower than using FI, providing incorrect cumulative antibiograms. Previous studies analyzed fewer organisms with a limited number of antimicrobial agents rather than all reported antimicrobial agents [13,14]. This is the first study to evaluate all reported antimicrobial agents for seven major clinical pathogens and analyze these data using WHONET.

In this study, the susceptibility rates determined by FI were significantly lower than those by TI for many antimicrobial agents, particularly when evaluating gram-negative organisms. Multi-drug-resistant organisms were also lowered when calculated by FI than by TI. Kohlmann et al. reported that the resistance rates were lower when repetitive isolates were removed, and the resistance rates of early isolates were lower than those of late isolates [14]. The CLSI guideline also states that the main problem in calculating TI is that resistance estimates are heavily weighted toward findings in patients with multiple cultures, long hospital stays, treatment failures, or complicated clinical histories [4]. Several factors may lead to lower susceptibility rates calculated by TI than by FI, such as variable specimens with the same resistance pathogens from the same patients, multiple specimens from the same site of the patients, and the emergence of antimicrobial resistance due to antimicrobial treatment. Moreover, clinicians are more likely to order follow-up cultures for isolates showing resistance. In our study, the TI/FI ratio was highest for *A. baumannii* (3.4), indicating that an average of 3.4 replicates of *A. baumannii* were isolated from one patient. *E. faecalis* showed the lowest TI/FI ratio (1.2) among the study organisms. Our study results of a positive relationship between TI/FI ratios and a larger gap in susceptibility rates support that calculated susceptibility rates are higher when duplicate isolates are removed (Table 2) [14]. The main purpose of reporting cumulative antibiograms is to guide empirical therapy in patients when final culture reports are not available. Therefore, results from patients with long hospital stays and duplicate isolates are not useful for guiding the treatment of patients who require empirical therapy. Low and misreported susceptibility rates determined by TI interfere with patient treatment.

**Figure 1:**
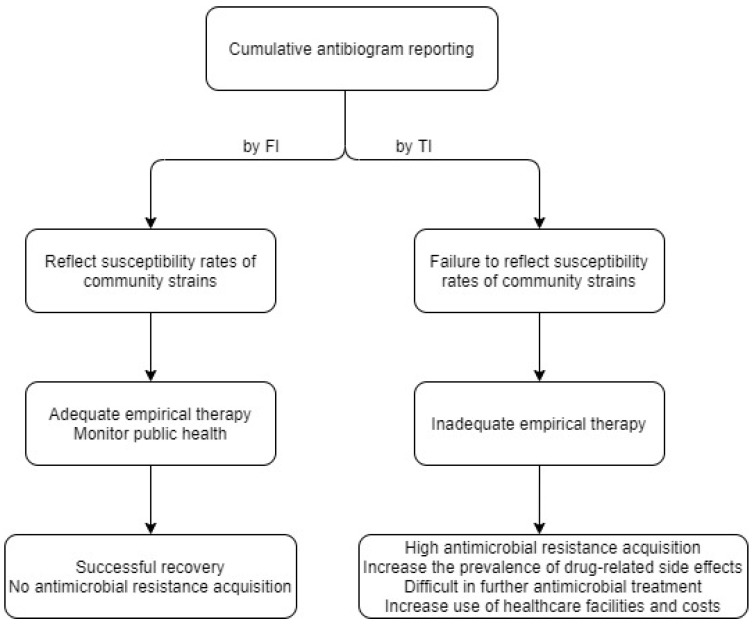
Scenarios when cumulative antibiograms are calculated by FI and by TI. FI, first isolate per patient; TI, total isolate.

The main critical principles of antimicrobial treatment include choosing an adequate antimicrobial agent at the correct dose for the clinical patients’ conditions and performing treatment for the appropriate duration [15]. Resistance can emerge when antimicrobial agents are misused. From a clinical perspective, it is important to begin appropriate antimicrobial therapy as early as possible, even in the absence of specific information on the pathogen. Empirical therapy, or preemptive antimicrobial therapy, is used to treat patients. Antibiograms vary widely between countries and local regions within countries [16,17]. It is well-known that local epidemiological antibiograms are required to prescribe more suitable antimicrobial agents considering the local flora while decreasing the emergence of antimicrobial resistance [12]. The emergence of antimicrobial resistance due to increased and inappropriate antibiotic treatment reduces the treatment options and overall efficacy of antimicrobials [16]. Inappropriate overuse of antimicrobial agents promotes antimicrobial resistance, increases the prevalence of drug-related side effects, and increases the use of healthcare facilities, all of which result in unnecessary costs [18,19,20,21,22]. Therefore, the clinical microbiology laboratory of each hospital should report appropriate cumulative antibiograms to guide adequate empirical therapy, although there are some technical difficulties when these results are determined by FI (Figure 1).

CLSI guideline M39-A4 recommends that only the first isolate of a given species per patient per analysis period should be included, irrespective of the body site, antimicrobial susceptibility profile, or other phenotypic characteristics when calculating the cumulative antibiogram [4]. The World Health Organization (WHO) also commented that it is important to express an antibiogram within a defined human population rather than using the number of isolates [23]. Despite these guidelines, it remains difficult for clinical microbiologists to calculate the cumulative antibiogram based on the first isolates. Software specializing in cumulative antibiograms such as WHONET or the R package AMR can be used [24]. WHONET is a free Windows-based database software developed for managing and analyzing microbiology data by the WHO collaborating center for surveillance of antimicrobial resistance [7]. This specialized program can be used to analyze antimicrobial susceptibility tests. Before analysis using WHONET, users must first match all codes of their own hospital, including antimicrobial agents, species name, patient information, ward information, and departments to those in the WHONET program. This matching process is the main factor making the program difficult to use. After matching, users can analyze data from their own hospitals based on various criteria such as isolation, by the first isolate only, and by the most resistant result for each antibiotic according to the latest international guidelines, including CLSI and European Committee on Antimicrobial Susceptibility Testing (EUCAST), which are difficult to perform using basic programs such as Excel. Studies involving clinicians in local areas performed by in-person conferencing may support the use of WHONET.

This study had some limitations. First, we used data from a single hospital, which may not be representative of the susceptibility situation in Korea. Second, the numbers of some organisms were small. For *Enterococcus* species, considerable differences may be observed between the two methods if the number of isolates is higher. If we collect and analyze susceptibility data for several years, the number of isolates can be increased.

## 5. Conclusions

In conclusion, when comparing cumulative antibiograms by FI with by TI using real-world data, a large gap was observed in critical species of hospital infection control. Even if FI calculation is difficult in practice, antibiograms must be calculated using FI for proper preemptive antimicrobial therapy and infection control.

## Figures and Tables

**Table 1:** Antimicrobial agents used in this study.

Organism	Antimicrobial Agets
*Escherichia coli*	AMK, AMP, AMX, AZT, CEZ, CIP, CTX, ERT, FEP, GEN, IMI, TAZ, TGC, TMX
*Acinetobacter baumannii*	AMX, AZT, CIP, COL, CTX, FEP, GEN, IMI, LEV, MER, MIN, PIP, TAZ, TGC, TMX
*Klebsiella pneumoniae*	AMK, AMP, AMX, AZT, CEZ, CIP, CTX, ERT, FEP, GEN, IMI, TAZ, TGC, TMX
*Pseudomonas aeruginosa*	AMK, AZT, CIP, CTX, FEP, GEN, IMI, LEV, MER, PIP, TAZ
*Staphylococcus aureus*	CIP, CLN, ERY, GEN, OXA, PEN, RIF, TEI, TET, TMX, VAN
*Enterococcus feacalis*	AMP, CIP, GEN, LNZ, PEN, Q/D, STR, TEI, TET, VAN
*Enterococcus faecium*	AMP, CIP, GEN, LNZ, PEN, Q/D, STR, TEI, TET, VAN

AMK, amikacin; AMP, ampicillin; AMX, amoxicillin/clavulanic acid; AZT, aztreonam; CEZ, cefazolin; FEP, cefepime; CTX, cefotaxime; TAZ, ceftazidime; CIP, ciprofloxacin; CLN, clindamycin; COL, colistin; ERT, ertapenem; ERY, erythromycin; GEN, gentamicin; IMI, imipenem; LEV, levofloxacin; LNZ, linezolid; MER, meropenem; MIN, minocycline; OXA, oxacillin; PEN, penicillin; PIP, piperacillin; Q/D, quinupristin/dalfopristin; RIF, rifampin; STR, streptomycin; TEI, teicoplanin; TET, tetracycline; TGC, tigecycline; TMX, trimethoprim/sulfamethoxazole; VAN, vancomycin.

**Table 2:** Number of isolates evaluated by FI and TI and TI/FI ratio.

Type	Organism	Number of Isolates (Proportion)		TI/FI	Mean of (S%^FI^–S%^TI^) *
		FI	TI		
Gram-negative	*Escherichia coli*	1824 (24.2%)	2692 (21.4%)	1.5	3.7
	*Acinetobacter baumannii*	480 (6.4%)	1611 (12.8%)	3.4	14.5
	*Klebsiella pneumoniae*	662 (8.8%)	1306 (10.4%)	2.0	8.3
	*Pseudomonas aeruginosa*	407 (5.4%)	953 (7.6%)	2.3	7.9
Gram-positive	*Staphylococcus aureus*	649 (8.6%)	1364 (10.9%)	2.1	4.4
	*Enterococcus feacalis*	323 (4.3%)	394 (3.1%)	1.2	0.3
	*Enterococcus faecium*	211 (2.8%)	313 (2.5%)	1.5	0.1

FI, first isolate per patient; TI, total isolate; S%, percentage of susceptibility rate * Average differences in susceptibility rates for each antimicrobial agent between FI and TI.

**Table 3:** Differences in susceptibility rates between calculated from FI and TI for each antimicrobial agent.

Type	Organism	Antimicrobial Agents with Different Susceptibility Rates between TI and FI		
		FI > TI		FI ≤ TI
		*p* < 0.05	*p* > 0.05	
Gram-negative	*Escherichia coli*	AMP, AMX, CEZ, TAZ, CTX, FEP, AZT, ERT, IMI, AMK, GEN, CIP	TMX, TGC	
	*Acinetobacter baumannii*	PIP, AMX, TAZ, CTX, FEP, IMI, MER, GEN, CIP, LEV, TMX, TGC	AZT, MIN	COL
	*Klebsiella pneumoniae*	AMX, CEZ, TAZ, CTX, FEP, AZT, ERT, IMI, GEN, CIP, TMX, TGC	AMP, AMK	
	*Pseudomonas aeruginosa*	PIP, TAZ, FEP, AZT, IMI, MER, CIP, LEV	CTX, AMK, GEN	
Gram-positive	*Staphylococcus aureus*	OXA, GEN, CIP, CLN, ERY, TET	PEN, RIF	TMX, VAN, TEI
	*Enterococcus feacalis*		GEN, STR, LNZ	PEN, AMP, CIP, VAN, TEI, Q/D, TET
	*Enterococcus faecium*		CIP, VAN, TEI	PEN, AMP, GEN, STR, LNZ, Q/D, TET

FI, first isolate per patient; TI, total isolate; AMK, amikacin; AMP, ampicillin; AMX, amoxicillin/clavulanic acid; AZT, aztreonam; CEZ, cefazolin; FEP, cefepime; CTX, cefotaxime; TAZ, ceftazidime; CIP, ciprofloxacin; CLN, clindamycin; COL, colistin; ERT, ertapenem; ERY, erythromycin; GEN, gentamicin; IMI, imipenem; LEV, levofloxacin; LNZ, linezolid; MER, meropenem; MIN, minocycline; OXA, oxacillin; PEN, penicillin; PIP, piperacillin; Q/D, quinupristin/dalfopristin; RIF, rifampin; STR, streptomycin; TEI, teicoplanin; TET, tetracycline; TGC, tigecycline; TMX, trimethoprim/sulfamethoxazole; VAN, vancomycin.

**Table 4:** Prevalence of multi-drug resistant organisms calculated by FI and TI.

Organism	FI	TI	*p*
CREC	1.6% (29/1824)	2.1% (57/2692)	0.227
CRKP	10.0% (66/662)	13.1% (171/1306)	0.046
CRAB	64.0% (307/480)	82.2% (1324/1611)	<0.0001
CRPA	40.5% (165/407)	48.6% (463/953)	0.006
MRSA	62.4% (405/649)	69.8% (952/1364)	0.001
VRE *	45.9% (97/211)	50.5% (158/313)	0.302

* Only *Enterococcus feacium* were included. FI, first isolate per patient; CREC, carbapenem-resistant *Escherichia coli*; CRKP, carbapenem-resistant *Klebsiella pneumoniae*; CRAB, carbapenem-resistant *Acinetobacter baumannii*; CRPA, carbapenem-resistant *Pseudomonas aeruginosa*; MRSA, methicillin-resistant *Staphylococcus aureus*; VRE, vancomycin-resistant *Enterococcus*.

## Supplementary Materials

The following are available online at https://www.mdpi.com/1010-660X/56/10/507/s1, Figure S1: Susceptibility rates of each antimicrobial agent of FI and TI on study isolates.
