# Supplementary material for: Importance of Susceptibility Rate of ‘the First’ Isolate: Evidence of Real-World Data
Source: Medicina (Kaunas). 2020 Sep 28;56(10):507. doi: 10.3390/medicina56100507 (PMC7600976; doi:10.3390/medicina56100507)
Supplement: Supplementary file 1 [file medicina-56-00507-s001.pdf]

Supplement. Susceptibility rates of each antimicrobial agents of FI and TI on study isolates

Organisms	Antimicrobial agents	Susceptibility (%)			*p* value
		FI	TI	FI - TI	
Escherichia coli	Ampicillin	29.8	26.6	3.2	0.0186
	Amoxicillin/Clavulanic acid	69.2	65.1	4.1	0.0041
	Cefazolin	60.4	54.5	5.9	0.0001
	Ceftazidime	64.8	58.8	6.0	<0.0001
	Cefotaxime	63.6	57.3	6.3	<0.0001
	Cefepime	66.9	60.6	6.3	<0.0001
	Aztreonam	66.0	60.0	6.0	<0.0001
	Ertapenem	99.6	98.6	1.0	0.0009
	Imipenem	98.9	97.7	1.2	0.003
	Amikacin	99.3	98.6	0.7	0.0281
	Gentamicin	71.8	68.3	3.5	0.012
	Ciprofloxacin	56.1	51.5	4.6	0.0024
	Trimethoprim/Sulfamethoxazole	63.7	61.3	2.4	0.1026
	Tigecycline	99.6	99.4	0.2	0.3589
Klebsiella pneumoniae	Ampicillin	1.1	0.6	0.5	0.2301
	Amoxicillin/Clavulanic acid	61.1	48.7	12.4	<0.0001
	Cefazolin	56.8	43.8	13	<0.0001
	Ceftazidime	60.7	48.6	12.1	<0.0001
	Cefotaxime	59.8	46.8	13.0	<0.0001
	Cefepime	62.8	51.2	11.6	<0.0001
	Aztreonam	61.9	49.7	12.2	<0.0001
	Ertapenem	95.9	90.4	5.5	<0.0001
	Imipenem	94.0	87.1	6.9	<0.0001
	Amikacin	97.6	95.9	1.7	0.0535
	Gentamicin	82.3	76.0	6.3	0.0014
	Ciprofloxacin	68.3	57.1	11.2	<0.0001
	Trimethoprim/Sulfamethoxazole	71.8	66.5	5.3	0.0170
	Tigecycline	85.5	81.6	3.9	0.0299
Acinetobacter baumannii	Piperacillin	31.2	13.4	17.8	<0.0001
	Ampicillin/Sulbactam	36.0	16.3	19.7	<0.0001
	Ceftazidime	34.8	15.7	19.1	<0.0001
	Cefotaxime	27.7	12.7	15.0	<0.0001
	Cefepime	37.7	17.1	20.6	<0.0001
	Aztreonam	3.5	2.6	0.9	0.2948
	Imipenem	42.3	21.5	20.8	<0.0001
	Meropenem	39.2	17.8	21.4	<0.0001
	Gentamicin	49.4	30.7	18.7	<0.0001
	Ciprofloxacin	36.7	16.1	20.6	<0.0001
	Levofloxacin	36.9	16.1	20.8	<0.0001
	Trimethoprim/Sulfamethoxazole	54.8	39.4	15.4	<0.0001
	Colistin	98.1	98.5	-0.4	0.5389
	Minocycline	89.8	88.6	1.2	0.4631
	Tigecycline	87.9	82.5	5.4	0.0049
Pseudomonas aeruginosa	Piperacillin	57.5	46.8	10.7	0.0003
	Ceftazidime	64.6	55.3	9.3	0.0015

	Cefotaxime	2.0	1.6	0.4	0.6035
	Cefepime	65.7	56.8	8.9	0.0022
	Aztreonam	52.1	44.4	7.7	0.0092
	Imipenem	65.8	51.3	14.5	<0.0001
	Meropenem	66.7	52.6	14.1	<0.0001
	Amikacin	85.3	84.1	1.2	0.5761
	Gentamicin	78.9	77.6	1.3	0.5963
	Ciprofloxacin	64.6	51.5	13.1	<0.0001
	Levofloxacin	61.0	47.9	13.1	<0.0001
	Colistin	99.3	98.8	0.5	0.4077
Staphylococcus aureus	Penicillin G	6.3	5.0	1.3	0.2286
	Oxacillin	36.6	30.2	6.4	0.0041
	Gentamicin	63.6	54.3	9.3	0.0001
	Rifampin	95.8	94.4	1.4	0.1841
	Ciprofloxacin	54.1	46.3	7.8	0.0011
	Trimethoprim/Sulfamethoxazole	95.4	97.1	-1.7	0.0508
	Clindamycin	53.2	44.6	8.6	0.0003
	Erythromycin	48.2	41.3	6.9	0.0035
	Vancomycin	100.0	100.0	0.0	-
	Teicoplanin	99.5	99.7	-0.2	0.4866
	Tetracycline	65.5	57.0	8.5	0.0003
Enterococcus faecalis	Penicillin G	88.5	89.3	-0.8	0.7342
	Ampicillin	98.1	98.5	-0.4	0.6786
	Gentamicin-High	43.8	43.3	0.5	0.8932
	Streptomycin-High	85.7	85.5	0.2	0.9396
	Ciprofloxacin	77.9	78.2	-0.3	0.9231
	Linezolid	99.1	98.7	0.4	0.6128
	Vancomycin	99.4	99.5	-0.1	0.8565
	Teicoplanin	99.4	99.5	-0.1	0.8565
	Quinupristin/Dalfopristin	0.0	0.0	0.0	-
	Tetracycline	13.6	16.2	-2.6	0.3327
Enterococcus faecium	Penicillin G	6.2	7.1	-0.9	0.6872
	Ampicillin	6.2	7.1	-0.9	0.6872
	Gentamicin-High	52.9	54.3	-1.4	0.7528
	Streptomycin-High	89.9	90.4	-0.5	0.8504
	Ciprofloxacin	2.1	1.7	0.4	0.7399
	Linezolid	97.1	97.5	-0.4	0.7804
	Vancomycin	54.1	49.5	4.6	0.3020
	Teicoplanin	70.7	68.4	2.3	0.5759
	Quinupristin/Dalfopristin	89.9	90.8	-0.9	0.7314
	Tetracycline	77.5	80.6	-3.1	0.3904

Abbreviation: FI, first isolate per patient; TI, total isolate
